# Developing consensus measures for global programs: lessons from the Global Alliance for Chronic Diseases Hypertension research program

**DOI:** 10.1186/s12992-017-0242-8

**Published:** 2017-03-15

**Authors:** Michaela A. Riddell, Nancy Edwards, Simon R. Thompson, Antonio Bernabe-Ortiz, Devarsetty Praveen, Claire Johnson, Andre P. Kengne, Peter Liu, Tara McCready, Eleanor Ng, Robby Nieuwlaat, Bruce Ovbiagele, Mayowa Owolabi, David Peiris, Amanda G. Thrift, Sheldon Tobe, Khalid Yusoff, On behalf of the GACD Hypertension Research Programme, Amir Attaran, Anniza de Villiers, Amber Featherstone, Jamie Forrest, Robert Kalyesubula, Julius Kamwesiga, Andre P. Kengne, Paul Camacho Lopez, Edward Mills, Barbara Mukasa, Katherine Muldoon, Jean-Claude Tayari, Sanni Yaya, Ng Kien Keat, Patricio Lopez, Juan Lopez Casas, Tara McCready, Martin McKee, Eleanor Ng, Robby Nieuwlaat, Ariffin Omar Zainal, Khalid Yusoff Salim Yusuf, Norman Campbell, Kajiru Kilonzo, Peter Liu, Marion Marr, Sheldon Tobe, Karen Yeates, Xiangxian Feng, Feng He, Stephen Jan, Xian Li, Ching-Ping Lin, Jun Ma, Yuan Ma, Graham MacGregor, Caryl Nowson, Haijun Wang, Yangfeng Wu, Lijing Yan, Jianhui Yuan, Jing Zhang, Jane Goudge, Chodziwadziwa Kabudula, Felix Limbani, Nkosinathi Masilela, Nokuzola Myakayaka, Margaret Thorogood, Francesc Xavier Gómez-Olivé, Simin Arabshahi, Clara Chow, Roger Evans, Rohina Joshi, Kartik Kalyanram, Kamakshi Kartik, Ajay Mahal, Pallab Maulik, Brian Oldenburg, Michaela Riddell, Velandai Srikanth, Oduru Suresh, Kavumpurathu Thankappan, Sathish Thirunavukkarasu, Nihal Thomas, Amanda G. Thrift, Ravi Varma, Gari Clifford, Stephane Heritier, Stephen Jan, Rohina Joshi, Stephen MacMahon, Pallab Maulik, Anushka Patel, David Peiris, Dorairaj Prabhakaran, Devarsetty Praveen, Stephen Jan, Pallab Maulik, Anushka Patel, Dorairaj Prabhakaran, Anthony Rodgers, Abdul Salam, Simon Thom, Ruth Webster, Claire Johnson, Anand Krishnan, Sailesh Mohan, Bruce Neal, Dorairaj Prabhakaran, K. Srinath Reddyl, Roopa Shivashankar, Thout Sudhir, Sarah Faletoese, Merina Ieremia, Marj Moodie, Bruce Neal, Arti Pillay, Jimaima Schultz, Junior Siitia, Wendy Snowdon, Arleen Sukhu, Christina Ulberg, Satupaitea Viali, Jacqui Webster, Antonio Bernabe-Ortiz, María Kathia Cárdenas, Francisco Diez-Canseco, Robert H. Gilman, J. Jaime Miranda, Vilarmina Ponce-Lucero, Katherine Sacksteder, Kingsley Apusiga, Richard Cooper, Joyce Gyamfi, Michael Ntim, Olugbenga Ogedegbe, Jacob Plange-Rhule, Cynthia Binanay, Gerald Bloomfield, Allison De Long, Eric Finkelstein, Valentin Fuster, Joseph Hogan, Jemima Hoine Kamano, Carol Horowitz, Tom Inui, Sylvester Kimaiyo, Claire Kofler, Diana Menya, Violet Naanyu, Jackson Rotich, Rajesh Vedanthan, Eric Velazquez, Martin Were, Federico Augustovski, Andrea Beratarrechea, Jing Chen, Jacquelyn Dolan, Jiang He, Vilma Irazola, Marie Krousel-Wood, Katherine Mills, Rosana Poggio, Adolfo Rubinstein, Lizheng Shi, Larry Webber, Rufus Akinyemi, Oyedunni Arulogun, Mulugeta Gebregziabher, Samantha Hurst, Bruce Ovbiagele, Mayowa Owolabi, Ezinne Uvere, Salina Waddy, Stephanie Warth

**Affiliations:** 1 Department of Medicine, School of Clinical Sciences at Monash Health, Monash University, Melbourne, Australia; 2 School of Nursing, Faculty of Health Science, University of Ottawa, Ottawa, Canada; 3 Queen Mary University of London, London, UK; 4 Universidad Peruana Cayetano Heredia, Lima, Peru; 5 The George Institute for Global Health - India, Hyderabad, India; 6 The George Institute for Global Health - Sydney, Sydney, Australia; 7 South African Medical Research Council, Cape Town, South Africa; 8 University of Ottawa Heart Institute, Toronto, Canada; 9 Population Health Research Institute, Hamilton, Canada; 10 Medical University of South Carolina, Charleston, USA; 11 Department of Medicine, University of Ibadan, Ibadan, Nigeria; 12 The George Institute for Global Health, University of Sydney, Sydney, Australia; 13 Sunnybrook Health Sciences Center, Toronto, Canada; 14 UniversitiTeknologi MARA, Selangor, Malaysia; 15 UCSI University, Selangor, Malaysia

**Keywords:** Implementation, Consensus Measures, Implementation Context, Hypertension, Low and middle income countries

## Abstract

**Background:**

The imperative to improve global health has prompted transnational research partnerships to investigate common health issues on a larger scale. The Global Alliance for Chronic Diseases (GACD) is an alliance of national research funding agencies. To enhance research funded by GACD members, this study aimed to standardise data collection methods across the 15 GACD hypertension research teams and evaluate the uptake of these standardised measurements. Furthermore we describe concerns and difficulties associated with the data harmonisation process highlighted and debated during annual meetings of the GACD funded investigators.

With these concerns and issues in mind, a working group comprising representatives from the 15 studies iteratively identified and proposed a set of common measures for inclusion in each of the teams’ data collection plans. One year later all teams were asked which consensus measures had been implemented.

**Results:**

Important issues were identified during the data harmonisation process relating to data ownership, sharing methodologies and ethical concerns. Measures were assessed across eight domains; demographic; dietary; clinical and anthropometric; medical history; hypertension knowledge; physical activity; behavioural (smoking and alcohol); and biochemical domains. Identifying validated measures relevant across a variety of settings presented some difficulties. The resulting GACD hypertension data dictionary comprises 67 consensus measures. Of the 14 responding teams, only two teams were including more than 50 consensus variables, five teams were including between 25 and 50 consensus variables and four teams were including between 6 and 24 consensus variables, one team did not provide details of the variables collected and two teams did not include any of the consensus variables as the project had already commenced or the measures were not relevant to their study.

**Conclusions:**

Deriving consensus measures across diverse research projects and contexts was challenging. The major barrier to their implementation was related to the time taken to develop and present these measures. Inclusion of consensus measures into future funding announcements would facilitate researchers integrating these measures within application protocols. We suggest that adoption of consensus measures developed here, across the field of hypertension, would help advance the science in this area, allowing for more comparable data sets and generalizable inferences.

**Electronic supplementary material:**

The online version of this article (doi:10.1186/s12992-017-0242-8) contains supplementary material, which is available to authorized users.

## Background

The need to enhance global health research that can inform action on pressing health issues such as chronic diseases, infectious diseases and maternal and child health has prompted transnational partnerships among researchers and research funders. These partnerships can accelerate a critical mass of research directed at a common health issue, foster new alliances and networks among researchers that strengthen the overall research endeavour and its likelihood for success, and provide a common basis for decision making and advocacy using the best available evidence. A critical enabler to achieving these aims is the development of common metrics to maximize learning across multiple research projects.

There are two predominant models of global research collaborations. The first model provides a means to answer common research questions such as identification of common risk factors and disease burden that require large comparative multi-centre studies. Examples include collaborative projects such as the INTERHEART/INTERSALT [1, 2], World Health Organization MONICA project [3] and The Diabetes Attitudes, Wishes and Needs (DAWN) study [4]. These research studies are often undertaken in diverse settings. However, teams typically share a common study design, measurement instruments and methodologies. In these initiatives, data may be collected either contemporaneously or in sequence, but there is an underlying aim to facilitate robust comparisons by using pooled data to compare effect sizes.

The second utilises partnerships to facilitate program funding and policy development. Examples include the Global Fund to Fight AIDS, Tuberculosis and Malaria [5]; GAVI, The Vaccine Alliance [6]; Global Alliance for the Prevention of Obesity and Related Chronic Diseases [7], and the Peers for Progress network [8]. These funding programs tend to support research to determine the magnitude of the problem, develop and implement interventions and, in the case of low-and middle income countries (LMICs), build research and workforce capacity. Projects may be funded as a result of a request for applications (RFA) specific to a research area or broad research question. These RFAs tend to elicit projects with a diverse set of interventions and methods and as such data variables and collection methods may not be consistent across studies limiting the ability to perform cross site outcome analysis and evaluation of implementation strategies.

Established in 2010, [9], the Global Alliance for Chronic Diseases (GACD) [10, 11], is an example of a transnational partnership of health research funding agencies with aims and processes more similar to the second model described above (see Table 1 for description of GACD- Organisational, funding and research network processes). The broader aim of all funded research through the GACD [10], is to lessen the burden of multiple non-communicable diseases (NCDs) in LMICs by developing interventions which are amenable to scaling up and which incorporate disease specific outcomes to assess efficacy [12]. Implementation science, defined as “what works for whom and under what contextual circumstances, and is it scalable in equitable ways?” [13] has been a basis for developing all of GACD’s funding opportunities: This orientation provides the basis for the GACD to achieve larger global impacts as research teams examine how varying contexts (e.g. health care financing, governance, health human resource capacity, accessibility of health care etc.) influence interventions and their scalability; and how existing implementation gaps can be closed and health inequities reduced.

**Table 1:** Global Alliance for Chronic Diseases (GACD) - Organisational, funding and research network processes [10, 11]

The GACD member agencies are National public funding agencies that primarily fund health research in their own countries. These agencies have come together as a global alliance to contribute to and support infrastructure and research programmes under the auspices of the GACD through finance and management.
GACD alliance members agree on joint research priorities and fund world-class research, fostering collaboration of research programmes between low-and middle income countries and high income countries to fight chronic diseases. Alliance members issue joint requests for applications (RFAs) on a regular basis on topics in strategic focus areas.
Responses to RFAs undergo rigorous peer review through Alliance member’s existing funding processes, although alliance members are moving towards joint peer review by all member agencies. To date, this model has been piloted on a small scale on two of the previous funding calls, and rollout to all agencies is expected for 2017. While this peer-review panel makes recommendations for funding, funding decisions are ultimately made by each of the GACD member agencies, and they are the bodies who award and administer all research funds.
The research teams that receive funding as part of a GACD research programme form a community of researchers and funding agency representatives under the banner of the GACD Research Network (GRN). Through the network, members have the opportunity to participate in joint activities in order to share information and develop common approaches to their research. The Research Network meets annually at the GACD Annual Scientific Meeting, with additional conference calls throughout the year. The joint activities take the form of a number of Working Groups, which are formed and chaired by researchers who choose to work together on common themes across their projects. The collaborative efforts of the GACD Research network and its Working groups are supported by the GACD Secretariat, which is based in London, UK.
Current member agencies of GACD (as of December 2016):
• Argentinian Ministry of Science and Technology (MINCYT), Argentina
• National Health and Medical Research Council (NHMRC), Australia
• São Paulo Research Foundation (FAPESP), Brazil
• Canadian Institutes of Health Research (CIHR), Canada
• Chinese Academy of Medical Sciences (CAMS), China
• Research & Innovation DG, European Commission, EU
• Indian Council of Medical Research (ICMR), India
• Agency for Medical Research and Development (AMED), Japan
• National Institute of Medical Sciences and Nutrition Salvador Zubirán, Mexico - funding available through Conacyt
• South African Medical Research Council (SA MRC), South Africa
• Health Systems Research Institute, Thailand
• Medical Research Council (MRC), United Kingdom
• National Institutes of Health (NIH), United States

**Fig. 1:**
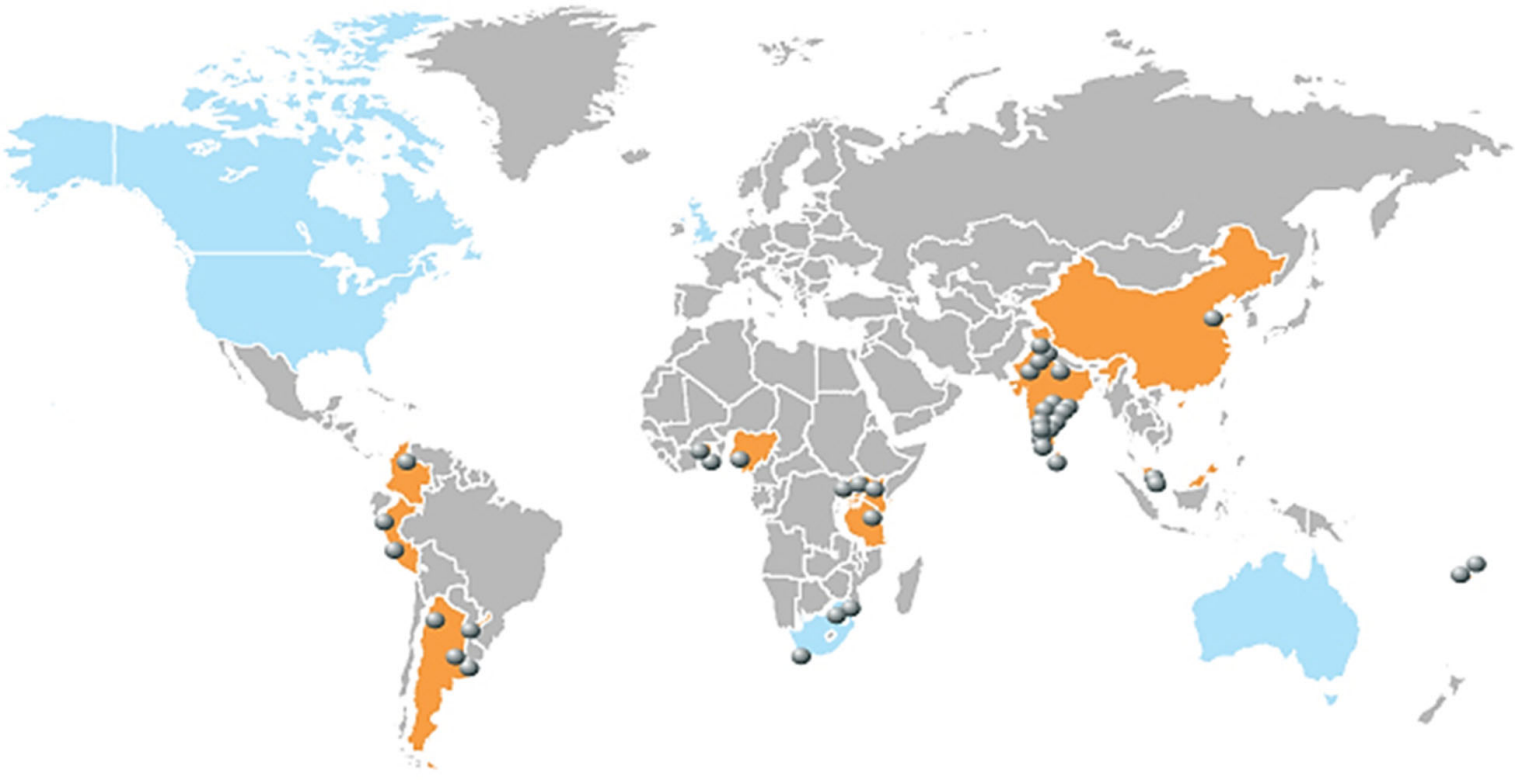
GACD Hypertension funding agencies and location of each data collection site. Countries/Regions in *blue* indicate original GACD funding partners for the GACD Hypertension (HT) programme. Countries in *orange* indicate low-middle income (LMIC) partner countries for HT research. Circles indicate LMIC location of research project

**Fig. 2:**
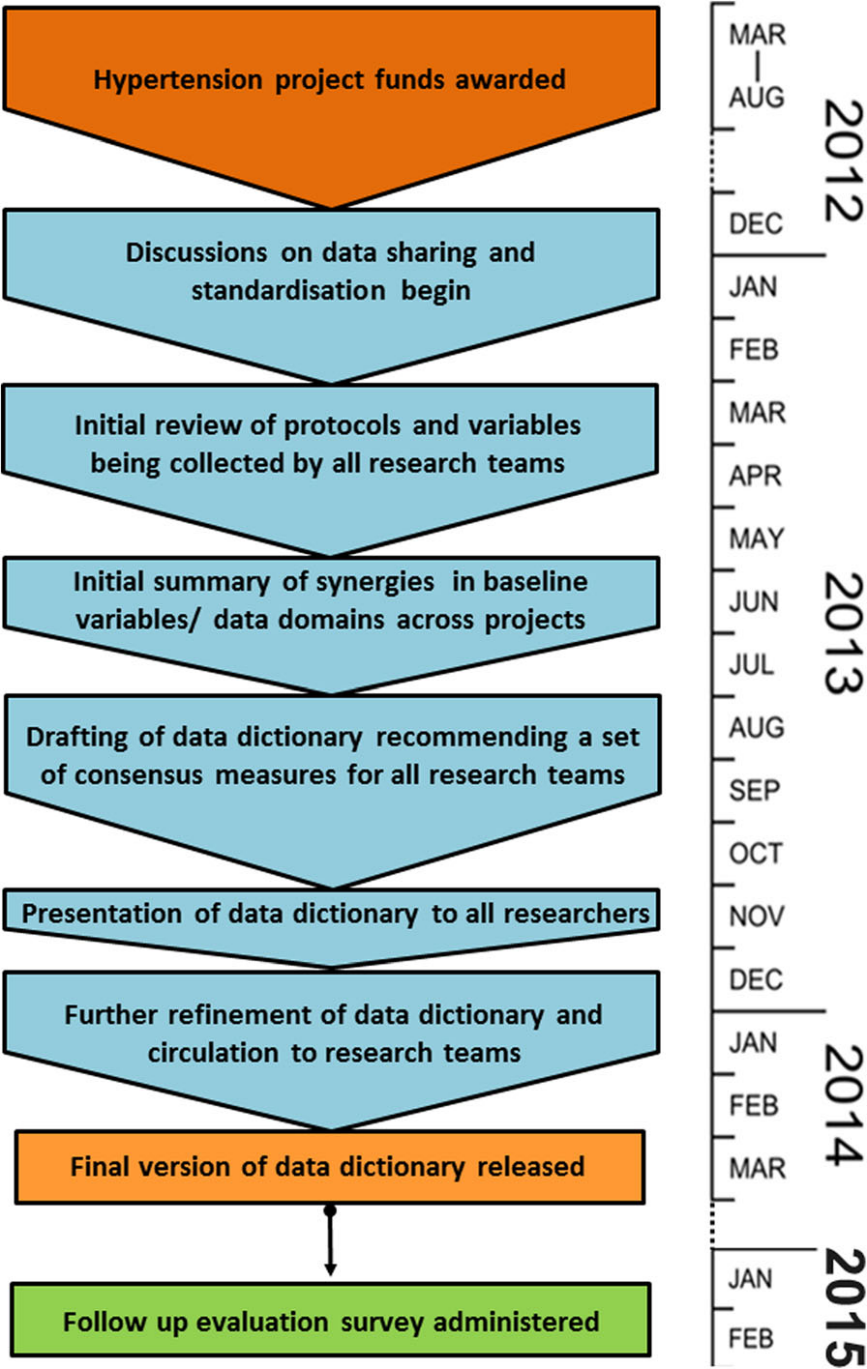
Data harmonisation process and evaluation timeline

The initial GACD funding round (see Table 1 for GACD organisational, funding and research network processes [10]), described here, focused on prevention, management and control of hypertension and emphasised implementation science relevant and suitable to LMIC settings, and in Aboriginal communities within Canada and Australia (here after referred to as LMICs) [11]. Fifteen projects across sixteen countries received more than US$23 million committed over five years in response to this first round of GACD Hypertension RFAs (Fig. 1). National funding agencies announced successful projects over a 6 to 9 month period during 2012, (Fig. 2) which resulted in projects having different starting dates.

The GACD Board asked that a common set of clinical outcomes be used to enable comparisons of outcomes across funded projects. Thus, common text was included in the Request for Applications (RFAs) from each agency regarding expectations of research teams to share data, to develop approaches to standardise data collection and, wherever feasible, use these standardised approaches in their respective projects [11]. However, a recent systematic review of interventions aimed at multiple risk factors for primary prevention of cardiovascular disease (CVD) in LMICs revealed only 13 eligible studies. The authors found that the pooled estimates of effect size for risk factor changes was questionable due to heterogeneity of data [14], and there was no investigation of the role of contextual factors in explaining this heterogeneity. In another review of lifestyle interventions to lower blood pressure (BP) and assess the multiple intervention effect on BP in LMIC, geographical or country specific context heterogeneity was limited to ethnicity [15]. Recently statistical modelling and data pooling, such as that used by the NCD Risk Factor Collaboration to estimate global trends in blood pressure, have informed the global epidemiology of blood pressure [16]. However, several limitations to this approach, including scarcity of primary data from LMICs and inconsistent protocols and measurement devices for collecting primary data over time and between countries (digital vs standard mercury sphygmomanometer), likely resulted in wider uncertainties of estimates for some regions and countries.

The aims of this paper are: a) to describe the experience of identifying, developing and encouraging the use of a common set of standard indicators that would enable comparisons of clinical outcomes across the 15 funded hypertension projects and also enhance understanding of implementation strategies to prevent and control hypertension in LMICs and b) to evaluate/examine the uptake and utility of recommendations for data harmonization by funded hypertension research teams.

## Methods

See Table 2 and Fig. 2 for timeline outlining the process of data harmonisation, data dictionary development and implementation evaluation.

**Table 2:** Timeline of data harmonisation, data dictionary development and evaluation

Date of activity	Activity undertaken
March–August, 2012	Successful Hypertension GACD Programme awardees announced.
December,2012–February, 2013	Discussion group formed at GACD ASM.
	Data Standardisation Working Group proposed and agreed upon.
March, 2013	Data Standardisation Working Group formally constituted.
March–August, 2013	Scoping exercise to identify potential consensus variables and summarise data across eight domains for all Hypertension Programme projects.
August–November, 2013	Data dictionary drafted as a recommended set of consensus measures based on previous scoping exercise and summary steps
November, 2013	Data Standardisation Working group presents recommendations for common measures to be adopted at 2013 GACD ASM.
December, 2013–February, 2014	Further refinement of draft data dictionary based on feedback received at 2013 GACD ASM.
February, 2014	Final version of consensus data dictionary released
February, 2015	Follow-up survey conducted to assess level of adoption of recommended measures.
April–November, 2015	Analysis of implementation of data dictionary by teams

### Preliminary discussion of data harmonisation and sharing

Discussions concerning the potential for both data sharing and data harmonisation and standardisation were initiated at the 2012 GACD Joint Technical Steering Committee (JTSC), (now known as the GACD Research Network (GRN)), Annual Scientific Meeting (ASM) in Ottawa (8–11 December 2012) (Table 2 and Fig. 2). This took place 6 months after the announcement of the successful hypertension projects. Representatives from all hypertension research projects and funding agencies were present. During this meeting a discussion group, comprising representatives interested in data harmonisation from some of the 15 research teams, was formed to discuss shared data opportunities, identify potential issues relating to the logistics and value of data sharing and to make recommendations to the JTSC Ottawa meeting for further discussion of data harmonisation and sharing opportunities, the logistics of data management, value of data sharing to all teams and ethical considerations.

A working group whose members were representatives from 8 of the 15 research teams and of the GACD funding agencies was formally convened in early 2013 by teleconference attendance.

### Data sharing and harmonization process

As an initial step, the working group aimed to gain a greater understanding of the protocols and proposed measurements planned by each research team; to assess commonalities, beyond their joint focus on hypertension; and to use this information to establish standard variables and methodologies for a number of measures. This dataset would then serve as a framework for potential future cross-site analyses, and also as a reference source for research teams who were yet to establish their data collection methods.

Following this initial scoping survey of the 15 teams, working group members summarised data for eight domains comprising demographic, dietary, clinical and anthropometric, medical history, hypertension knowledge, physical activity, behavioural (smoking and alcohol), and biochemical domains; and then identified any existing concordance in measurement methods. Based on this initial analysis the working group prepared, and iteratively refined, a set of standardised variables using a modified (non-anonymous) Delphi technique [17]. This summary exercise was completed in August 2013 (Table 2 and Fig. 2).

At the 2013 GACD ASM, over a year after the initial JTSC (GRN) meeting (Table 2 and Fig. 2), the working group presented its recommendations for the common measures to be adopted in the form of the data dictionary (Additional file 1: Table S1). After further refinements based on feedback received at the meeting, the final version of the data dictionary was released to the research teams in February, 2014 (Table 2 and Fig. 2).

A follow-up survey was circulated in February 2015 (Table 2 and Fig. 2) to the 15 funded research teams asking whether or not they had included any of the consensus measures in their data collection, which measures they had included, and their reasons for including (already part of their planned protocol, to allow comparison with other groups, consensus measures were superior to planned measures) or not including (data collection imminent, not relevant to population/study, didn’t see value in comparing with other groups, IRB/permit constraints, financial/logistical constraints, unaware of consensus data dictionary) the consensus measures.

## Results

When opportunities and recommendations of the discussion group were initially shared with the JTSC (GRN), comprising all hypertension teams, at the 2012 Ottawa ASM doubts were raised about the value of joint data analysis, given that not all of the studies included population based data collection, and the interventions were diverse. Other concerns expressed were over (1) data ownership; (2) data sharing methodology; (3) the addition of variables not previously included in original protocols and potentially extraneous to the original study aims; and (4) ethical issues such as collecting data not directly related to the research question or adding protocols after the ethics approval process had been initiated. The ethical aspects of collecting data that would be shared with other researchers were vigorously debated and discussed. Researchers suggested that such activity would need to be included in participant information and consent forms. Some researchers believed that their study populations may have difficulty in understanding the concept of data sharing among teams and such activities may be a barrier to successful recruitment and furthermore create suspicion among study participants about what would happen to their data and how it would be used. Despite the aforementioned concerns which we discuss further in this article, the consensus of the representatives attending the 2012 ASM was to form a working group to progress data harmonisation among the 15 research teams.

The initial scoping exercise, by the formal working group during March – August, 2013 (see Table 2 and Fig. 2) yielded mixed results. Although there appeared to be large potential for data standardisation, there were discrepancies in both the types of variables collected and the methods and protocols to measure common variables (such as blood pressure (BP)). There was considerable variation in the degree of overlap in measures among research groups, but the greatest consistency was observed in planned measurements relating to BP, current smoking status, anthropometry, physical activity and medical history (previous diagnosis of diabetes/hypertension/stroke or hypertension medication).

Differences in methodologies were associated with the planned intervention, the study setting (health care/clinical setting vs community based), the target population (e.g. school children, adults, policy-makers), cross cultural considerations (particularly in the domains of nutrition and tobacco use) as well as relevance of the measures to the main research question (s) of each research team.

Particular difficulties were experienced in (1) identifying validated measures (e.g. diet and physical activity), which were relevant across diverse ethno-cultural settings and pertinent for populations from varying socioeconomic positions; and (2) balancing the use of well validated measures with what was logistically possible and pertinent for studies that were being undertaken in health care settings ranging from rural and remote primary care clinics to tertiary care hospitals. Teams also believed that the addition of unplanned variables would likely increase participant burden and possibly affect recruitment and participation. There were also teams with interventions that focused on policy rather than behavioural outcomes changes. These teams were concerned about the pertinence of clinical measures for their studies.

Despite these concerns, the GACD hypertension data dictionary was developed (Additional file 1: Table S1) based on the initial scoping survey of the 15 funded teams and the iterative process undertaken by the working group to summarise and identify appropriate measures for inclusion in the data dictionary. It comprised a total of 67 consensus measures across eight common domains (Table 3 and Additional file 1: Table S1): demographic; dietary; clinical and anthropometric; medical history; hypertension knowledge; physical activity; behavioural (smoking and alcohol), and biochemical.

**Table 3:** Data domains within data dictionary

Domain	Description
Demographic	Participant age, gender and information relating to household size and income.
Diet	Variables collecting information on salt intake, and meat/vegetable consumption.
Clinical/Anthropometry	WHO STEPS [30] blood pressure protocol, and basic anthropometric measurements.
Personal Medical History	Participant’s history of CVD and diabetes.
Knowledge of HTN	Participant’s knowledge and awareness of hypertension.
Physical activity	Details concerning patient’s level of regular physical exercise.
Behavioural	
Smoking	Level of tobacco use
Alcohol	Level of alcohol consumption.
Biochemical	24 h Urine and blood glucose measurement from WHO STEPS biochemical core [30].

One team did not respond to the follow up survey in February 2015. Of the 14 responding teams, only two teams were including more than 50 consensus variables, five teams were including between 25 and 50 consensus variables and four teams were including between 6 and 24 consensus variables. One team did not provide details of the variables being collected and two of the 14 teams indicated that they did adopt any of the standardised methods. Additional file 1: Table S1 details the number of teams collecting each of the consensus measures. Measurements relating to the demographic domain (date of birth, highest education, sex), clinical/anthropometry domain (blood pressure, pulse rate, anthropometry), behavioural (smoking) domain (current smoking status), physical activity domain (physically active for more than 30 min five times/week, how much time spent walking or cycling) and medical history (previous diagnosis of diabetes/hypertension/stroke or hypertension medication) were being collected by eight or more of the 11 teams (Additional file 1: Table S1). Of the two teams not including any of the measures, one had already submitted applications for ethics approval and data collection was imminent by the time the consensus variables were available and the other team, assessing cost effectiveness of salt reduction intervention in the Pacific Islands, determined the standardised measures were not relevant for their study.

All eleven teams who included consensus variables in their protocols, stated that the consensus variables they were using were already included in their original protocols, two of the teams indicated that they changed some of their original data variables to align with the consensus measures, stating the consensus measures were superior to those initially planned. Five teams expressed support for the consensus measures to enable data comparability with other teams.

Dietary measures proved particularly difficult to standardise given global dietary diversity (e.g. patterns of fruit, vegetable and protein intake) and this was reflected in the number of teams using the proposed dietary consensus measures. Six teams planned to ask if salt was added to meals during cooking and five teams planned to ask about adding salt to food after cooking. Four teams planned to ask about fruit, vegetable and protein consumption and only three teams planned to ask about dairy intake.

## Discussion

The GACD Hypertension programme consists of 15 teams investigating a multitude of interventions with the overall aim of improving the detection, treatment, and monitoring of people with hypertension in resource-limited settings. This study identified and described the processes undertaken to harmonise the data collection of the 15 research teams funded under the GACD hypertension programme. Furthermore we highlight issues and difficulties related to this harmonisation process across the 15 teams.

Each team is contributing to the evidence-base within its own particular context. However, the collaboration of GACD teams would enhance this by enabling analyses of baseline prevalence of hypertension and associated risk factors across settings, enabling the broader evaluation of intervention modalities and implementation issues, and ensuring that setting and context are considered when assessing individual and population outcomes. The approach taken in the development of the consensus measures was initially driven by clinical outcome measures and known risk factors commonly used in systematic reviews and meta-analysis. Whilst this approach was appropriate for reporting baseline prevalence, analysis of implementation impact also requires the inclusion of contextual variables, such as barriers and facilitators, which may impact the intervention outcomes and affect scalability. The development of contextual measures for implementation science is still a nascent area of development [18].

### Why develop consensus measures?

As can be seen from the GBD study [19] and studies such as the DAWN study [4], consensus measures, collected using consistent methodologies, can improve overall estimates of impact. Taking the lead from other areas of study (e.g. infectious disease, injury prevention, and cancer control), the importance of a “case definition” is crucial for consistent identification of people with hypertension and enhances comparability across studies. Thus consensus measures can be particularly valuable when comparing interventions, implementation methods and individual and population outcomes across different settings and contexts.

By sharing data that includes common outcome measures, research teams could potentially generate new hypotheses, be able to answer additional research questions to their original project focus, and conduct analyses with enhanced sample sizes and power. It could also foster new collaborations and maximise use of each project’s data. An example of such a collaboration between members of the GACD hypertension programme identified, using the behaviour change wheel framework [20], the ability to achieve behaviour change across regions and between those responsible for health care delivery (clinicians, non-physician health workers and policy makers) differed greatly [21]. The process of data standardisation has also led to an increased understanding of what data are necessary to answer the research questions of the GACD programme as a whole, and the opportunity for each research team to critically evaluate their planned methodologies.

### Weighing up accuracy and precision against feasibility of consensus measures

In order to compare the impact of implementation strategies across settings, we would typically propose the consistent measurement of outcomes across sites. The variety of methods to measure hypertension (e.g. automated BP vs manual sphygmomanometer) as well as variability in clinical definitions of hypertension [22] exacerbates difficulties in ensuring data consistency. While there were consensus measures available, teams were faced with pragmatic issues such as the need to measure blood pressure using locally available equipment in district health centres. This approach was in keeping with the implementation science focus, with teams aiming to examine the scale up of interventions under local conditions. This orientation required that teams strike a balance between using clinical measures that are consistent with usual care practices (and available equipment) versus using more refined measures (and newly acquired equipment). While the latter provides better measurement precision, it also introduces an intervention intrusion that may limit the accurate assessment of intervention scalability.

### Challenges to developing/implementing consensus measures

#### Consistency of methods

Consensus measures should not only collect the same information but also, where practical, collect it using the same methodologies. In the case of blood pressure, various methodologies exist from manual to automatic readers, from one reading to three readings, ambulatory or office bound. Thus consistency across various cultural and geographical settings needs to be balanced between the desire for scientific precision and real world implementation.

#### Ethical issues

In discussions of lessons learned, the ethical aspects of sharing data were identified. Data sharing intentions should be clearly stated in any information shared with the participants of the study and consent is necessary for data sharing to occur. Therefore, consensus measures and an understanding of the extent of data sharing should be in place prior to ethical approval being sought for the individual projects.

Further to this, ethics committees often discuss the question of study relevance. Is it necessary and ethical to collect data which will not specifically contribute to the research question asked by the researchers? However, if through data sharing, greater value is extracted from an individual research project (and therefore from a participant’s involvement), there may exist an ethical imperative for this sharing of data. This should be made explicit at the outset through the inclusion of overarching questions that may be addressed via protocols using shared data.

The ethical question of “who owns the data” is one which should be negotiated and agreed upon by researchers, participants and global collaborative teams prior to any data collection and might be aligned with discussions of benefit and reciprocity.

The question of lack of reciprocity has been highlighted by van Panhuis et al. [23] as an ethical issue of data sharing for research involving LMICs. Benefits to populations as well as researchers, who contribute data to a collective analytic endeavour, must be clearly outlined and established at the outset. Such benefits might include health improvements for participating communities, and more equitable scientific outputs by participating researchers.

### Benefits of collecting consensus measures

Developing and collecting consensus measures can contribute better quality data to hasten global research efforts targeting prevention and management strategies for NCDs and their scale-up. Furthermore, developing global collaborations in which researchers operate independently and yet contribute cooperatively to inform the same research area can potentially minimise redundancies in data collection and implementation strategies.

Consensus measures collected consistently from different settings provide invaluable information for implementation strategies. Intervention outcomes to evaluate intervention effectiveness (“relative advantage”) [24] as well as information about context, setting and individual characteristics have been highlighted as important components of proposed frameworks to enhance implementation, such as the Consolidated Framework for Implementation Research [25]. The experience of the hypertension teams suggest that a larger set of comparative processes and outcomes can be examined than those traditionally examined in meta-analyses [21].

In addition, publication of these consensus measures may encourage new research teams that are not part of the GACD, to adopt these measures enabling them to contribute to the collective research effort on chronic disease prevention and management.

### Identifying gatekeepers and managers of consensus data

Collecting data and developing protocols and data formats for shared data can be complicated and time consuming. Few recipients of funds will have budgeted for this activity, if they have not been prompted to do so. Funding partners may need to develop data sharing policies and formal data sharing mechanisms to enable researchers to include such activities in their applications. The decision about who will be the gatekeepers of shared data; who will have access to the data and on what basis, need to be well established early to avoid potential conflicts or disagreements about the use of such data. This agreement should be obtained by members of the contributing research teams and authorship of any subsequent publications should have a clearly outlined policy and framework for all team members to follow. Issues of data access and ownership by Indigenous communities, such as those specified in the principles of ownership, control, access and possession [26, 27], also need to be taken into consideration.

### Who should guide the development of consensus measures and when should they be announced?

One option to ensure early adoption of consensus measures is for individual funding agencies to mandate what data and what related measurement methodologies should be used. However, this approach risks stifling innovative and novel research approaches, restricting investigation into the “known unknowns” or burdening research teams with the requirement to collect measures that may be irrelevant to their original research question. Alternatively, consensus measures could be developed as soon as funded projects are announced, with experts in the field recommending appropriate consensus measures (based on those already being proposed). This would enable teams to continue to pursue their original research hypothesis as well as contribute data to the greater collaboration. Importantly, this approach would potentially enable consistency of methodologies and definitions to be incorporated into protocols before submission to ethics review boards. With respect to the GACD, a number of the consensus measures developed for hypertension have applicability to other chronic diseases and we are encouraging the adoption of these measures by teams recently funded to address type 2 diabetes.

In addition to health-related outcomes, specifying requirements for some “higher-order” data about cultural, economic, policy and health system contexts to be collected would allow better learning about approaches to hypertension control that do or do not work across diverse contexts [13, 18, 28]. Shared learnings between groups under this funding arrangement can be realised through collaboration and networking between group members [21]. Such insights are an important goal for the implementation science agenda of the GACD programme overall.

### Health research funding agency protocols and timelines

The degree to which the GACD Hypertension Programme research teams adopted the set of consensus measures was influenced by several features of the GACD collaboration. Despite all GACD funding agencies including language on data sharing in their RFAs, clear direction and guidance on the likely additional outcomes of interest (beyond hypertension) were not available to teams when developing their research proposals. Successful projects were not announced by all funding agencies concurrently and funds were released to all successful research teams over a 6 to 9 month window. Therefore the first meeting of the JTSC (GRN) and the establishment of a core measure consensus group took place after some teams were well advanced with developing measurement tools, and submitting ethics applications. The consensus data dictionary was developed as an outcome of the first meeting of the JTSC (GRN) but the delay (~18 months) in the release of the data dictionary, meant that most teams were already commencing data collection when the data dictionary was provided. Ultimately only two of the fifteen GACD teams amended their study protocol to accommodate any consensus measures. However, a further 9 teams indicated that some of the consensus measures were already incorporated in their initial protocols.

This underscores the importance that data standardisation and data sharing issues be tackled early by consortia and that all funding partners and potential researchers should develop a common vision of future joint analysis activities from the outset.

## Conclusion

This paper has described the process of conceptualising and developing a set of consensus measures that would allow robust, relevant, and reliable comparisons to be made across projects and has outlined some of the challenges and questions to guide development of consensus measures in other global studies. Whilst standardising data collection methods and sharing the resultant datasets is an attractive proposition for both researchers and funding agencies alike, establishing a set of consensus measures requires a significant investment in effort from all parties, and substantial preparatory work. Implementation science will yield deeper insights more quickly if consensus measures are established for both health outcomes and contextual parameters. Relevant and workable data standardisation and sharing policies must be put in place to yield the potential benefits of collective efforts to analyse cross-project data. Policy trends towards increased open access to clinical trial data [29], make these consensus efforts timely and more achievable.

**Key recommendations:**

- ➢ Researchers and funders need to share a common vision for joint programme activities
- ➢ Clear and specific language on data standardisation and sharing should be included in the RFA.
- ➢ Funded teams should be brought together as soon as possible after funding announcements making use of communication technology to facilitate group contact.
- ➢ Consensus measures (both the outcome and context variables and the measurement approach) need to be developed as early as possible, preferably prior to ethics approval and participant recruitment.
- ➢ Adopt a pragmatic approach to balancing precision and direct comparability of common measures with the aims of implementation science including scalability.
- ➢ Measures of intervention context should be included in the consensus measures.
- ➢ Data sharing intentions should be included in informed consent documents.
- ➢ Make the consensus measures easily accessible so that others can utilise similar methods and approaches, and contribute to a data repository.

## Additional file

Additional file 1: Table S1. GACD hypertension consensus variables, suggested collection methods and number of teams using variable.(DOCX 36 kb)

## Abbreviations

DAWN: The Diabetes attitudes, wishes and needs program
GACD: Global alliance for chronic diseases
GBD: Global burden of disease
GRN: GACD research network (previously JTSC)
HIC: High-income Country
JTSC: Joint technical steering committee (now GRN)
LMIC: Low- and middle-income country
NCD: Non-communicable disease
RFA: Request for applications
