# Supplementary material for: Developing consensus measures for global programs: lessons from the Global Alliance for Chronic Diseases Hypertension research program
Source: Global Health. 2017 Mar 15;13:17. doi: 10.1186/s12992-017-0242-8 (PMC5353794; doi:10.1186/s12992-017-0242-8)
Supplement: Additional file 1: Table S1. — GACD hypertension consensus variables, suggested collection methods and number of teams using variable.(DOCX 36 kb) [file 12992_2017_242_MOESM1_ESM.docx]

### Supplementary Table 1

| **Domain** | **Variables** | **Specific methods** | **Source** | **# teams including** |
| --- | --- | --- | --- | --- |
| Demographic | Participant ID |  |  |  |
|  | Participant age | Actual (DOB or yrs), or estimate | *WHO STEPS core DEMOG [1]* | 8 |
|  | Sex | M/F | *WHO STEPS core DEMOG [1]* | 9 |
|  | Highest Education | # years at school or full time study | *WHO STEPS core DEMOG [1]* | 8 |
|  | Household size | # >18yrs sharing same kitchen (living in same household) | *WHO STEPS expanded DEMOG [1]* | 6 |
|  | Household Income | Last year average household earning | *WHO STEPS expanded DEMOG [1]* | 5 |
| Diet | Added salt before eating | Categorical frequency | [2] <http://www.fao.org/docrep/014/i1983e/i1983e00.pdf> | 6 |
|  | Added salt during cooking | Categorical frequency | [2] | 7 |
|  | Frequency/day salty food intake | # frequency | [2] | 4 |
|  | Salt consumption | Categorical | [2] | 3 |
|  | Salt consumption | # teaspoons | [2] | 2 |
|  | Fruit consumption | # days / week | *WHO STEPS core diet [1]* | 4 |
|  | Fruit servings on days eaten | # servings (std serving) | *WHO STEPS core diet [1]* | 4 |
|  | Vegetable consumption | # days /week | *WHO STEPS core diet [1]* | 4 |
|  | Vegetable servings | # servings (std serving) | *WHO STEPS core diet [1]* | 4 |
|  | Meals /week Fried vegetables | # meals/week | *4Leaf survey [3]*  <https://4leafprogramdotcom.files.wordpress.com/2015/10/4leaf-pdf-survey-103015.pdf> | 4 |
|  | Meals /week meat/poultry | # meals/week | *4Leaf survey [3]* | 4 |
|  | Meals /week fish | # meals/week | *4Leaf survey [3]* | 4 |
|  | Meals /week other protein (legumes, nuts etc) | # meals/week | *4Leaf survey [3]* | 4 |
|  | Times /week dairy | # times/week | *4Leaf survey [3]* | 3 |
|  | Times /week deep fried foods | # times/week | *4Leaf survey [3]* | 4 |
|  | Oil/fat usually used for cooking(meal prep) |  | *WHO STEPS- expanded diet [1]* | 4 |
| Clinical/Anthropometry | Arm circumference cuff size | Cuff size (S, M, L, XL) |  | 5 |
|  | Systolic BP | Mean of 2^nd^/3^rd^ reading | *WHO STEPS Protocol recommended [1]* | 10 |
|  | Diastolic BP | Mean of 2^nd^/3^rd^ reading | *WHO STEPS Protocol recommended [1]* | 10 |
|  | Heart Rate (pulse) | Mean of 2^nd^/3^rd^ reading | *WHO STEPS Protocol recommended [1]* | 9 |
|  | Height | cm (stadiometer if possible) | *WHO STEPS Protocol recommended [1]* | 9 |
|  | Weight | Kg (digital scale if possible) | *WHO STEPS Protocol recommended [1]* | 9 |
|  | Waist circumference | cm (constant tension tape if possible) | *WHO STEPS Protocol recommended [1]* | 6 |
|  | Hip Circumference | cm (constant tension tape if possible) | *WHO STEPS Protocol recommended [1]* | 5 |
| Personal Medical History | History/ knowledge of hypertension | Ever been told by doctor or HCW of raised BP | *WHO STEPS core HT [1]* | 8 |
|  | Hypertension Medication | in past 2 weeks | *WHO STEPS core HT [1]* | 8 |
|  | History/ knowledge of Diabetes | Ever been told by doctor or HCW of diabetes | *WHO STEPS core HT [1]* | 8 |
|  | History/knowledge of CVD | Ever been told by doctor or HCW of heart problems | *WHO STEPS core HT [1]* | 7 |
|  | History of CVD (coronary artery bypass surgery | Ever had |  | 5 |
|  | History of CVD (coronary angioplasty) | Ever had |  | 5 |
|  | History of stroke | Ever been told by doctor or HCW of symptoms of stroke | *WHO STEPS core HT [1]* | 8 |
|  | History/knowledge of CKD | Ever been told by doctor or HCW of CKD | *WHO STEPS core HT [1]* | 7 |
| Knowledge of HTN | Knowledge of HTN | What does the term Hypertension mean? | [4] | 1 |
|  | Knowledge of HTN | How dangerous is hypertension to your health? | [4] | 1 |
|  | Knowledge of HTN | Would lowering high blood pressure improve a person’s health? | [4] | 0 |
|  | Awareness of HTN | Have you ever been told by a doctor or health care provider what your own blood pressure reading should be? | [4] | 0 |
|  | Knowledge of HTN | If told, what should your top number (systolic) be | [4] | 0 |
|  | Knowledge of HTN | If told, what should your bottom number (diastolic) be | [4] | 0 |
| Physical Activity | Physically active for>30 min 5 times/week |  | *WHO STEPS core Phy Act.[1]* | 8 |
|  | Time spent walking/ bicycle riding on typical day |  | *WHO STEPS core Phy Act. [1]* | 9 |
|  | Time spent sitting reclining on typical day |  | *WHO STEPS expanded Phy Act [1]* | 6 |
| Smoking | Smoking/chewing tobacco use | Current use smoking | [5] [http://www.itcproject.org/surveys International Tobacco Control Policy evaluation project](http://www.itcproject.org/surveys) | 10 |
|  | Smoking/chewing tobacco use | Current use smokeless | [5] | 7 |
|  | Smoking/chewing tobacco use | On average how often use (smoke or smokeless) | [5] | 5 |
|  | Smoking/chewing tobacco use | Ever consumed >100 | [5] | 4 |
|  | [Exposure to passive smoke from http://www.who.int/tobacco/media/en/jarvis.pdf](http://www.who.int/tobacco/media/en/jarvis.pdf) | Anyone smoking at home | [5] | 4 |
| Alcohol | Alcohol use | Ever consumed | *WHO STEPS Alcohol core [1]* | 6 |
|  | Alcohol use | Consumed (previous 12 months) | *WHO STEPS Alcohol core [1]* | 5 |
|  | Alcohol use | Past 12 months frequency of std drink | *WHO STEPS Alcohol core [1]* | 4 |
|  | Alcohol cessation | Stopped due to health advice | *WHO STEPS Alcohol core [1]* | 3 |
| Biochemical | Fasting blood glucose measure | Check fasting | *WHO STEPS biochemical core [1]* | 4 |
|  | Fasting blood glucose measure | Time of day | *WHO STEPS biochemical core [1]* | 4 |
|  | Fasting blood glucose measure | FBGL (mmol/L or mg/dL) | *WHO STEPS biochemical core [1]* | 4 |
|  | Fasting blood glucose measure | Check medication (insulin etc) | *WHO STEPS biochemical core [1]* | 4 |
|  | 24 hr Urine | 24 hr volume (ml) | *WHO STEPS biochemical core [1]* | 2 |
|  | 24 hr urine | assessment of complete sample | *WHO STEPS biochemical core [1]* | 2 |
|  | Na^+^ conc | Na^+^ estimate mEq/L/day | *WHO STEPS biochemical core [1]* | 2 |

**References**

1. World Health Organization: **The WHO STEPwise approach to noncommunicable disease risk factor surveillance (STEPS) -Instrument v. 3.0.**

2. Kennedy G, Ballard T, Dop M: **Guidelines for measuring household and individual dietary diversity.** Italy: Food and Agriculture Organization of the United Nations,; 2010.

3. 4Leaf Global LLC: **The 4Leaf Survey.** 2015.

4. Oliveria SA, Chen RS, McCarthy BD, Davis CC, Hill MN: **Hypertension Knowledge, Awareness, and Attitudes in a Hypertensive Population.** *Journal of General Internal Medicine* 2005, **20:**219-225.

5. **ITC Policy Evaluation Project - Surveys.** Canada; 2014.
